# Extracellular Vesicles Including Exosomes Regulate Innate Immune Responses to Hepatitis B Virus Infection

**DOI:** 10.3389/fimmu.2016.00335

**Published:** 2016-08-31

**Authors:** Takahisa Kouwaki, Yoshimi Fukushima, Takuji Daito, Takahiro Sanada, Naoki Yamamoto, Edin J. Mifsud, Chean Ring Leong, Kyoko Tsukiyama-Kohara, Michinori Kohara, Misako Matsumoto, Tsukasa Seya, Hiroyuki Oshiumi

**Affiliations:** ^1^Department of Immunology, Graduate School of Medical Sciences, Kumamoto University, Honjo, Chuo-ku, Kumamoto, Japan; ^2^Laboratory for Biologics Development, Research Center for Zoonosis Control, GI-CoRE Global Station for Zoonosis Control, Hokkaido University, Kita-Ku, Sapporo, Japan; ^3^Department of Microbiology and Cell Biology, Tokyo Metropolitan Institute of Medical Science, Kamikitazawa, Setagaya-ku, Tokyo, Japan; ^4^Section of Bioengineering Technology, Universiti Kuala Lumpur (UniKL) MICET, Melaka, Malaysia; ^5^Joint Faculty of Veterinary Medicine, Transboundary Animal Diseases Center, Kagoshima University, Korimoto, Kagoshima, Japan; ^6^Department of Microbiology and Immunology, Graduate School of Medicine, Hokkaido University, Kita-Ku, Sapporo, Japan; ^7^JST, PREST, Honjo, Chuo-ku, Kumamoto, Japan

**Keywords:** virus, innate immunity, exosome

## Abstract

The innate immune system is essential for controlling viral infection. Hepatitis B virus (HBV) persistently infects human hepatocytes and causes hepatocellular carcinoma. However, the innate immune response to HBV infection *in vivo* remains unclear. Using a tree shrew animal model, we showed that HBV infection induced hepatic interferon (IFN)-γ expression during early infection. Our *in vitro* study demonstrated that hepatic NK cells produced IFN-γ in response to HBV only in the presence of hepatic F4/80^+^ cells. Moreover, extracellular vesicles (EVs) released from HBV-infected hepatocytes contained viral nucleic acids and induced NKG2D ligand expression in macrophages by stimulating MyD88, TICAM-1, and MAVS-dependent pathways. In addition, depletion of exosomes from EVs markedly reduced NKG2D ligand expression, suggesting the importance of exosomes for NK cell activation. In contrast, infection of hepatocytes with HBV increased immunoregulatory microRNA levels in EVs and exosomes, which were transferred to macrophages, thereby suppressing IL-12p35 mRNA expression in macrophages to counteract the host innate immune response. IFN-γ increased the hepatic expression of DDX60 and augmented the DDX60-dependent degradation of cytoplasmic HBV RNA. Our results elucidated the crucial role of exosomes in antiviral innate immune response against HBV.

**Accession Number:**

Accession number of RNA-seq data is DRA004164 (DRA in DDBJ).

## Introduction

The innate immune response is the first line of defense against viral infection, and viral nucleic acids are recognized by pattern recognition receptors. Endosomal viral RNA and DNA are recognized by Toll-like receptor 3 (TLR3) and TLR7–9 (1). TLR7–9 require the MyD88 adaptor, whereas TLR3 requires the TICAM-1/Trif adaptor to initiate innate immune responses (1). Cytoplasmic double-stranded RNA (dsRNA) is recognized by RIG-I-like receptors (RLRs), RIG-I, and MDA5, which require the MAVS adaptor to trigger signaling (2). In contrast, cytoplasmic dsDNA is recognized by cGAS (3, 4) and IFI16 (5), which require the STING adaptor for the innate immune response. DDX60 is a cytoplasmic RNA helicase, which functions as a cell-type specific sentinel for the recognition and degradation of viral RNA and promotes the degradation of viral RNA (6, 7).

Extracellular vesicles (EVs) include microvesicles and exosomes. Microvesicles are released from the plasma membrane, and exosomes are released upon the exocytosis of multivesicular bodies; and CD9, CD63, and CD81 are markers of exosomes (8). An extensive body of evidence has shown that exosomes mediate intercellular communication (8) and also plays a crucial role for antiviral innate immune response. EVs including exosomes released from HCV-infected hepatocytes deliver viral RNA to dendritic cells, which is recognized by TLR3 and TLR7, resulting in type I interferon (IFN) production (9, 10). Moreover, exosomes deliver host microRNAs (miRs) and regulate the innate immune response to lipopolysaccharide (11). Recent studies indicate that not only exosomes but also microvesicles mediate intercellular communications (12).

Hepatitis B virus (HBV) is a major cause of hepatocellular carcinoma. RIG-I plays a dual role in the antiviral response to HBV in hepatocytes (13). RIG-I senses the ϵ region of viral pregenomic RNA, resulting in the production of type III IFN. In addition, the binding of RIG-I to the ϵ region counteracts the interaction between viral polymerase and pregenomic RNA, thereby suppressing viral replication (13). cGAS recognizes HBV dsDNA and induces an IFN-inducible gene, ISG56, to suppress viral replication in hepatocyte cell lines (14). However, the *in vivo* roles of RIG-I and cGAS in the innate immune response to HBV remain unclear. Type I IFN and type III IFN are well known to exhibit antiviral activities, but type II IFN (IFN-γ) also has antiviral activities against HBV, although the underlying mechanism is unclear (15, 16).

Hepatitis B virus infects humans and primates but not mice. Tree shrews (*Tupaia belangeri chinensis*) are small mammals that are susceptible to HBV infection, and the tree shrew animal model is a powerful tool for HBV research (17). In the present study, using the tree shrew animal model, we investigated the *in vivo* innate immune response to HBV. Our results demonstrated the crucial roles of EVs including exosomes *in vivo* during the innate immune response to HBV.

## Results

### HBV Induces Hepatic IFN-γ Expression *In Vivo*

**Figure 1:**
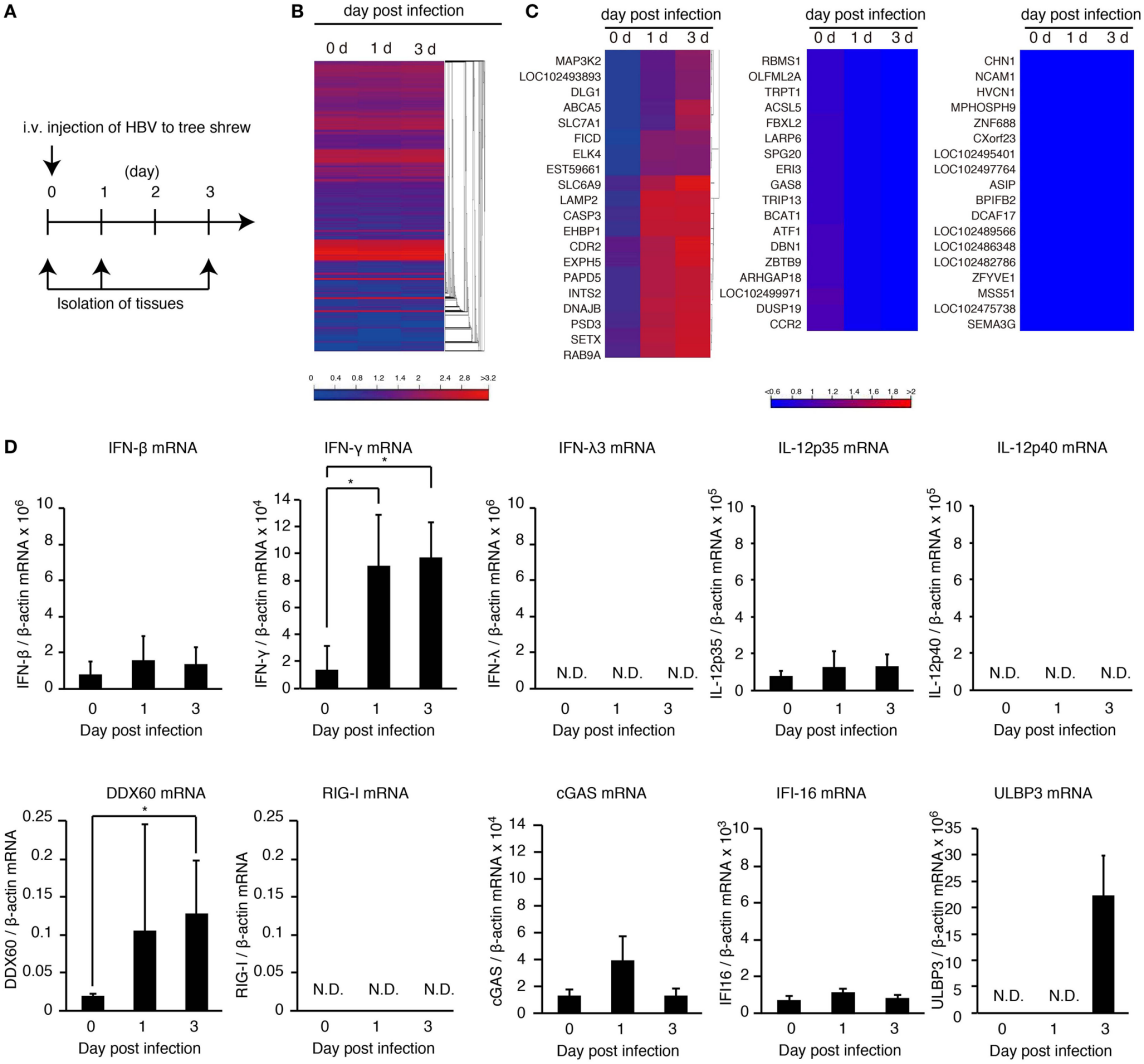
**HBV induces hepatic IFN-γ expression**. **(A)** Experimental procedure for infection and sampling. Tree shrews were infected intravenously with the HBV infectious particles. The livers were isolated on the day indicated. **(B,C)** Hierarchical clustering analysis of gene expression in the liver at 0, 1, and 3 days post-infection. Total RNA was extracted, and then RNA-seq analysis was performed using a next-generation sequencer. The data are representative of at least three independent experiments. The heat map of all genes was shown in **(B)**. The clustered regions, in which the expression of genes was upregulated (left panel), downregulated (center pane), and not changed by infection (right panel), were shown in **(C)**. **(D)** The gene expression levels in the liver of HBV-infected tree shrew were determined using RT-qPCR and was normalized against that of β-actin. Data are presented as mean ± SD (*n* = 4).

To investigate the *in vivo* innate immune response to HBV, HBV infectious particles were intravenously injected to tree shrews, and total RNA was isolated from the liver at 0, 1, and 3 days post-infection (Figure 1A). RNA-seq analysis was performed using a next-generation sequencer. A heatmap of all genes suggested that expression of most genes was not altered (Figure 1B and Figures S1A,B in Supplementary Material); however, there were several genes whose expression was affected by HBV injection (Figure 1C), and the induced expression of MAP3K2, RNase L, MDA5, and CD69 was detected (Figure 1C and Figure S1C in Supplementary Material). The IFN gene expression was not detected by RNA-seq analysis because of its low expression level.

To detect IFN-β, -γ, and -λ expression in tree shrew tissues, we performed RT-qPCR. HBV infection did not increase the expression of IFN-β in the liver, spleen, and kidney (Figure 1D and Figure S1D in Supplementary Material). In contrast, IFN-γ expression was specifically increased in the liver at 1 and 3 days post-infection (Figure 1D and Figure S1D in Supplementary Material). Considering that the adaptive immune response cannot produce IFN-γ within 1 day of primary infection, this early IFN-γ expression suggests that group 1 innate lymphoid cells (ILCs) are responsible for the early IFN-γ expression. In addition, HBV intravenous injection increased hepatic DDX60 expression but not RIG-I, cGAS, or IFI16 (Figure 1D).

**Figure 2:**
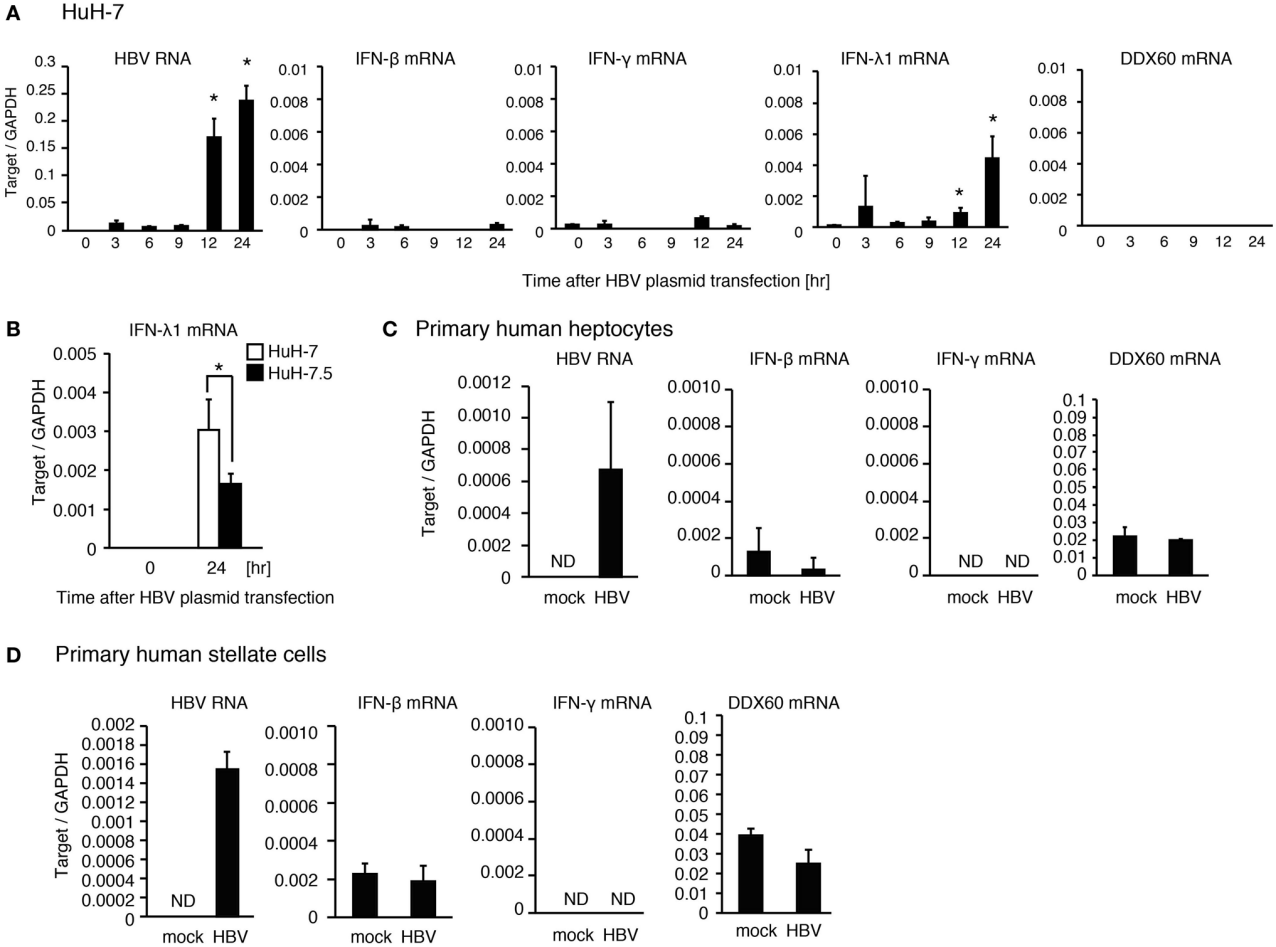
**The response of human hepatic cells to HBV**. **(A)** HuH-7 cells were transfected with pHBV plasmid, and RT-qPCR analysis was performed to determine the expression levels of HBV RNA, IFN-β, IFN-γ, IFN-λ1, and DDX60. Data are presented as mean ± SD (*n* = 3). **(B)** HuH-7 and HuH-7.5 cells were transfected with a plasmid carrying 1.4× HBV genome for 24 h. IFN-λ1 expression was determined using RT-qPCR and normalized to GAPDH. Data are presented as mean ± SD (*n* = 3). **(C)** Human primary hepatocytes were infected with HBV for 24 h, and the expression of the genes were determined by RT-qPCR. Data are presented as mean ± SD (*n* = 4). **(D)** Human primary hepatic stellate cells were infected with HBV for 24 h, and the expression of the genes was determined by RT-qPCR. Data are presented as mean ± SD (*n* = 4).

When hepatocyte cell lines, HepG2 and HuH-7, were transfected with a plasmid, pHBV, which carries 1.4× HBV genomic DNA and produces pregenomic RNA and all viral proteins, HBV RNA appeared at 3 h after transfection. However, the expression levels of DDX60 and IFN-γ did not increase until 24 h after transfection (Figure 2A and Figure S1E in Supplementary Material). As reported previously (13), HBV increased the RIG-I-dependent expression of IFN-λ in HuH-7 cells (Figure 2B). To further investigate the response of hepatic cells to HBV, we used primary hepatocytes and hepatic stellate cells. Infection of primary hepatocytes and hepatic stellate cells with HBV failed to increase IFN-γ and DDX60 expression (Figures 2C,D). These observations imply that hepatocytes and hepatic stellate cells cannot induce IFN-γ and DDX60 in response to HBV, and that non-parenchymal cells are required for hepatic IFN-γ and DDX60 expression.

### Extracellular Vesicles Induce the Expression of the NKG2D Ligands in Macrophages

**Figure 3:**
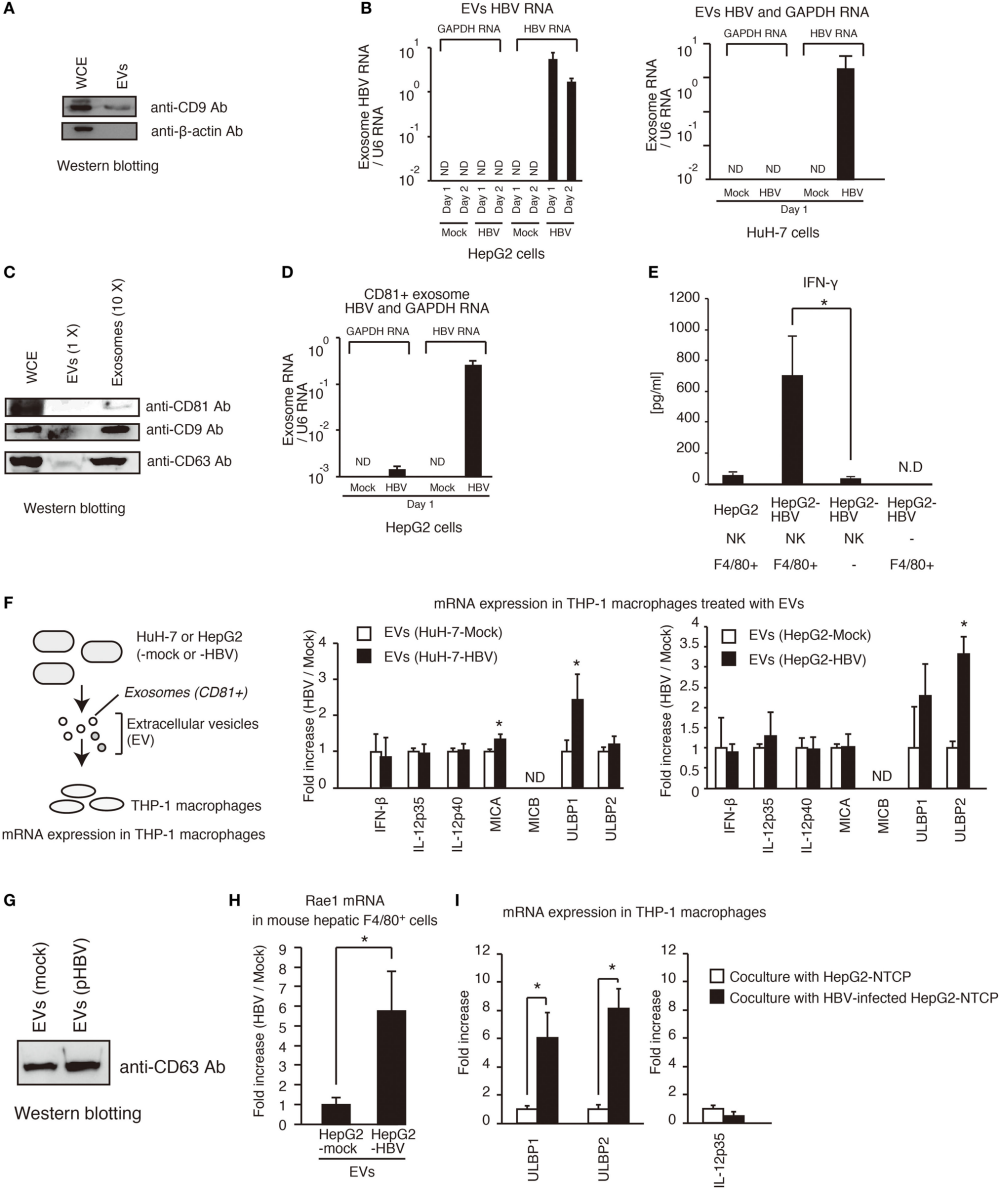
**Exosomes containing viral RNA induce NKG2D ligand expression in macrophages**. **(A)** HepG2 cells were seeded onto a 24-well plate and cultured for 24 h. EVs were isolated from 0.5 ml of cell culture medium using a polyethylene glycol method with exosome isolation kit (see Isolation of Exosomes in Experimental Procedures) and suspended with 150 μl of 1× SDS sample buffer. The 150 μl of whole cell extract (WCE) were prepared from cultured cells. The 10 μl of EVs and 10 μl of WCE were subjected to SDS-PAGE. CD9 and β-actin proteins were detected by western blotting with anti-CD9 antibody and anti-β-actin antibody. **(B)** Extracellular vesicles (EVs) released from HepG2 (left) or HuH-7 (right) transfected with pHBV were collected, and the total RNA was extracted. The RNA levels of HBV RNA and GAPDH mRNA were determined by RT-qPCR and normalized against that of U6 RNA. Data are presented as mean ± SD. **(C)** Exosomes were isolated from EVs released from HepG2 cells using anti-CD81 antibody beads. WCE, EVs, and 10× concentrated exosomes were subjected to SDS-PAGE, and the proteins were detected by western blotting. **(D)** The CD81^+^ exosomes were isolated from EVs with anti-CD81 microbeads. The RNA levels were determined as described in **(B)**. Data are presented as mean ± SD (*n* = 3). **(E)** Hepatic F4/80^+^ cells and hepatic NK cells were co-cultured with normal HepG2 cells or HepG2-T23 cells (HepG2-HBV), which stably express HBV, for 1 day. IFN-γ levels in the culture supernatants were determined using ELISA (*n* > 3). **(F)** EVs released from HuH-7 or HepG2 with or without HBV were added to PMA-treated THP-1 cells (THP-1 macrophages) for 24 h. The expression of mRNA in THP-1 macrophages was determined by RT-qPCR and normalized to GAPDH (*n* = 3). **(G)** HepG2 cells in six-well plates were transfected with mock or pHBV and were cultured for 24 h. EVs were isolated from 5 ml of culture medium and were suspended with 100 μl of PBS. The 5 μl of EVs were mixed with 5 μl of 2× SDS sample buffer and were subjected to SDS-PAGE. The proteins were detected by western blotting with anti-CD63 antibody. **(H)** EVs released from HepG2 with or without HBV were added to mouse hepatic F4/80^+^ cells for 24 h. The expression of mRNA in hepatic F4/80^+^ cells was determined by RT-qPCR and normalized to GAPDH (*n* = 3). **(I)** HepG2-NTCP cells were infected with HBV for 9 days and were subsequently co-cultured with THP-1 (transwell co-culture) for 3 days. The expression of mRNA in THP-1 macrophages was determined by RT-qPCR.

Hepatitis B virus infects hepatocytes but not non-parenchymal cells. Therefore, it was expected that hepatocytes would release the molecule that activates non-parenchymal cells. It should be noted that HCV-infected hepatocytes release the exosomes containing viral RNA, thereby leading to the activation of dendritic cells *via* TLRs (9), so we investigated whether HBV RNA is present in EVs including exosomes in a similar manner to HCV. First, we isolated EVs from cell culture medium of hepatocytes. We confirmed that EVs contained the CD9 protein, which is a marker of exosomes (Figure 3A). Interestingly, HBV RNA, but not host GAPDH mRNA, was detected within EVs released by pHBV-transfected hepatocytes (Figure 3B). Second, we isolated exosomes from HepG2 cell culture medium using anti-CD81 antibody beads, because exosomes released from HepG2 contain CD81 (18). Using the anti-CD81 beads, we could concentrate exosomes, and CD81, CD9, and CD69, which are a marker of the exosomes, were detected in the exosomes by western blotting (Figure 3C). As expected, HBV RNA was also detected in the CD81^+^ exosomes (Figure 3D), and viral DNA was also detected in the CD81^+^ exosomes (Figure S2A in Supplementary Material). In addition, CD81^+^ exosomes released from HBV-infected HepG2-NTCP cells contained HBV RNA (Figure S2D in Supplementary Material).

NK cells are the major group 1 ILCs in liver. In general, NK cells can produce IFN-γ (19), whereas Kupffer cell, which are hepatic macrophages, activates T and NK cells, resulting in IFN-γ production by T or NK cells (20). Hepatic NK cells isolated from mice failed to produce IFN-γ in the presence of HepG2-T23 cells, which stably express pHBV and produce HBV infectious particles (Figure 3E). Interestingly, hepatic NK cells produced IFN-γ in the presence of both HepG2-T23 and hepatic F4/80^+^ cells, which is a marker of macrophages (Figure 3E), suggesting the importance of macrophages. Therefore, we next, investigated whether EVs released from hepatocytes with pHBV affect macrophage function.

Extracellular vesicles were isolated from hepatocytes with or without pHBV. HBV transfection to hepatocytes barely affected the CD63 protein levels of EVs (Figures 3F,G). Interestingly, EVs released from hepatocytes with pHBV increased the mRNA expression of NKG2D ligands (MICA, ULBP1, ULBP2, and Rae1) in THP-1 macrophages or hepatic F4/80^+^ cells (Figures 3F,H). NKG2D ligands are known to elicit IFN-γ production from NK cells (21, 22). These data are correlated with upregulation of CD69, a NK cell activation marker, in tree shrew liver after HBV intravenous infection (Figure S1C in Supplementary Material). We confirmed that infection of HepG2-NTCP with HBV cells substantially increased the expression of ULBP1 and ULBP2 mRNA in co-cultured THP-1 macrophages, which was cultured using transwell co-culture system (Figure 3I).

**Figure 4:**
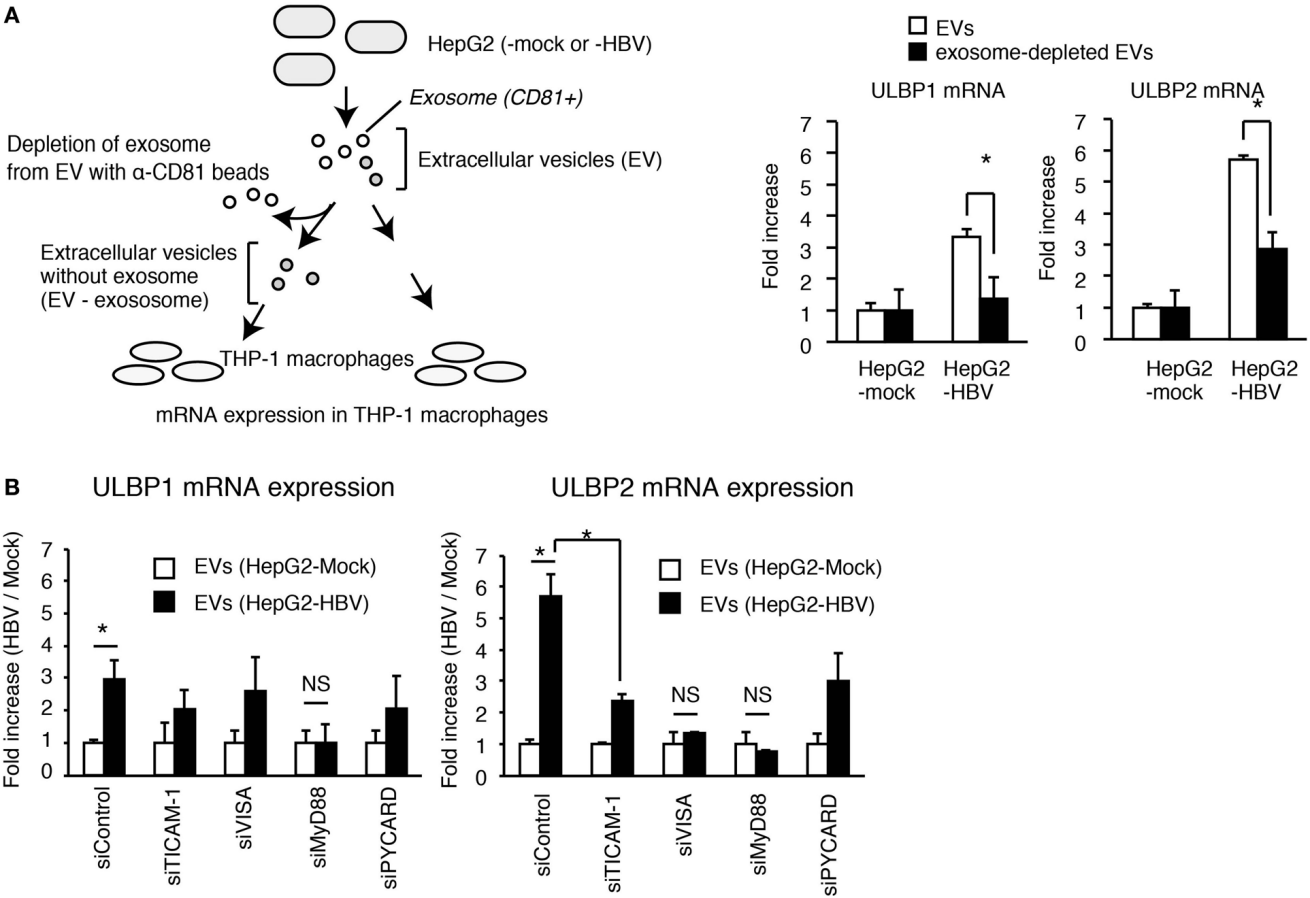
**The role of exosomes in the activation of PRRs**. **(A)** EVs released from HepG2 cells transfected with mock or pHBV was treated with or without anti-CD81 antibody beads (α-CD81 beads) and was subsequently added to THP-1 macrophages for 24 h. ULBP1 and ULBP2 mRNA expression in THP-1 macrophages were determined by RT-qPCR and normalized to GAPDH. Data are presented as mean ± SD (*n* = 3). **(B)** THP-1 macrophages were transfected with siRNA for mock, TICAM-1, VISA, MyD88, and PYCARD and were subsequently treated with EV from HepG2 with or without HBV for 24 h. ULBP1 and ULBP2 mRNA expression in THP-1 macrophages were determined by RT-qCR and normalized to GAPDH. Data are presented as mean ± SD (*n* = 3).

To assess the role of exosomes in NKG2D ligand expression, we performed a depletion assay. The depletion of CD81^+^ exosomes attenuated the EVs-mediated ULBP1 and ULBP2 expression in THP-1 macrophages (Figure 4A), indicating the importance of CD81^+^ exosomes. Interestingly, knockdown of MyD88 reduced EVs-mediated ULBP1 and ULBP2 expression, and ULBP2 expression was also reduced by TICAM-1 or VISA knockdown (Figure 4B). These results indicate that the CD81^+^ exosomes stimulate the MyD88, TICAM-1, and VISA pathways. Knockdown of PYCARD moderately decreased the expression of ULBP2 (Figure 4B); however, the ULBP2 expression was induced by TLRs and RLRs stimulation even in the absence of PYCARD stimulation (see below).

### microRNA (miR) within EVs Controls the Host Innate Immune Response

**Figure 5:**
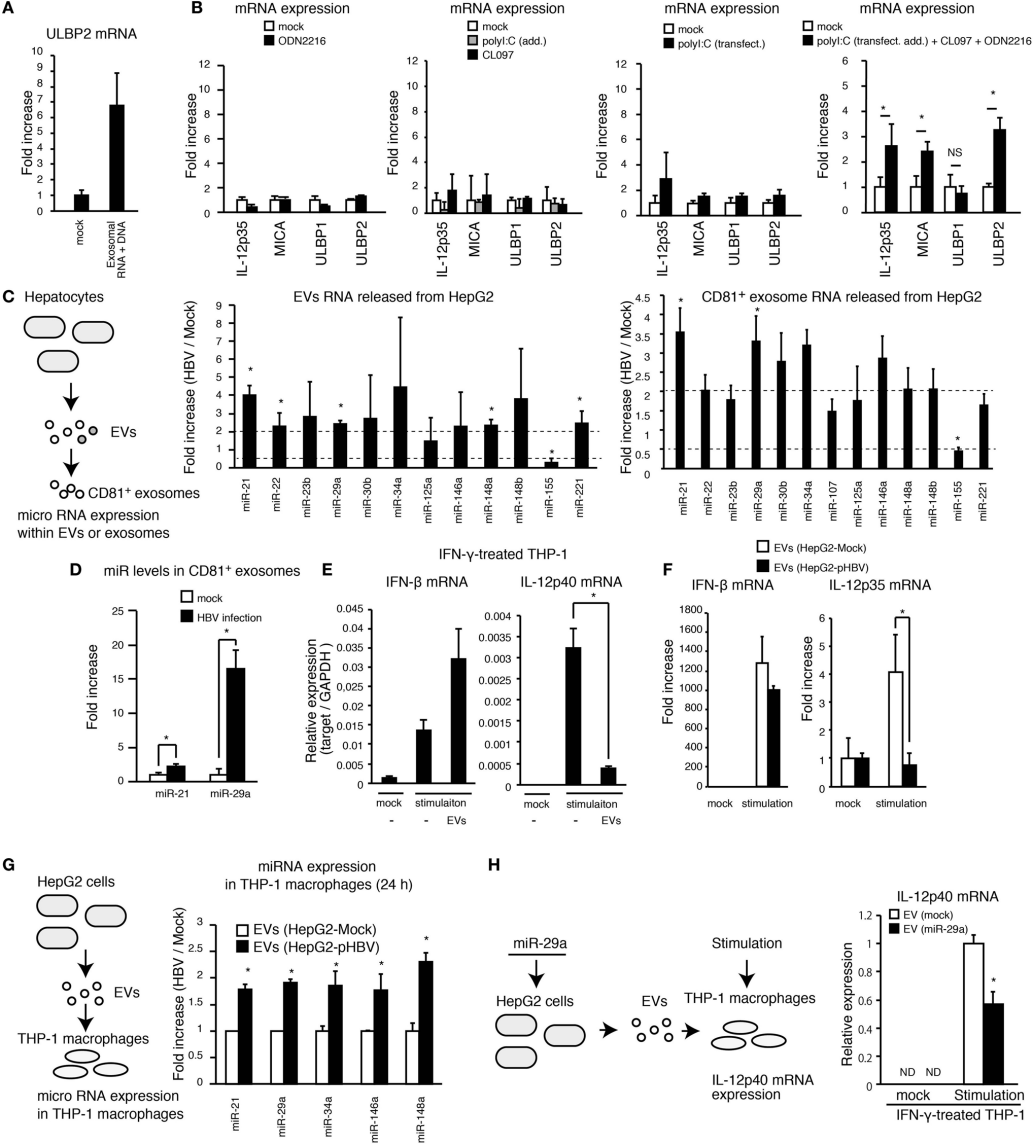
**Exosomes with miR attenuates the IL-12p35 mRNA expression**. **(A)** CD81^+^ exosomes were isolated from HepG2 with pHBV. Mock or 5 μg of exosomal RNA was added to THP-1 macrophages for 6 h, and then cells were transfected with mock or 1 μg of exosomal RNA and 1 μg of DNA for 24 h. ULBP2 mRNA expression was determined by RT-qPCR and normalized to GAPDH. **(B)** PMA-treated THP-1 cells were stimulated with 500 μM of ODN2216, 1 μg/ml of CL097, and/or polyI:C [transfection (2 μg/ml) and addition (100 μg/ml)] for 24 h. The expression of mRNA was determined by RT-qPCR. **(C,D)** RNA was extracted from EVs and CD81^+^ exosomes released from HepG2 with or without HBV **(C)** or HBV-infected HepG2-NTCP cells **(D)**, and the expression of miR was determined by RT-qPCR and normalized to U6 RNA level. Fold increase of miR expression was calculated by dividing miR level of HBV sample by that of mock. **(E)** THP-1 macrophages were treated with IFN-γ together with mock or EVs that were isolated from 1 ml of HepG2 cell culture medium for 24 h in a 24-well plate and were subsequently stimulated with 1 μg/ml of CL097 and polyI:C [transfection (2 μg/ml) and addition (100 μg/ml)] for 24 h. IFN-β and IL-12p40 mRNA expression was determined by RT-qPCR and normalized to GAPDH. **(F)** EVs were isolated from 1 ml of cell culture medium of HepG2 with or without pHBV. THP-1 macrophages were treated with EVs and were then simulated with 500 μM of ODN2216, 1 μg/ml of CL097, and polyI:C [transfection (2 μg/ml) and addition (100 μg/ml)] for 24 h in a 24-well plate. The expression of IFN-β and IL-12p35mRNA in THP-1 macrophages was determined by RT-qPCR and normalized to GAPDH. **(G)** EVs were isolated from 1 ml of cell culture medium of HepG2 cells. THP-1 macrophages were treated with EVs from HepG2 cells with or without pHBV for 24 h in a 24-well plate. Cells were washed twice with PBS, and total RNA was extracted from THP-1 cells. The expression of miR in THP-1 macrophages was determined by RT-qPCR. **(H)** HepG2 cells were transfected with miR-29a for 1 day, and EVs were subsequently isolated from HepG2 cell culture supernatant. THP-1 macrophages were treated with EVs for 1 day and then stimulated with 1 μg/ml of CL097 and polyI:C [transfection (2 μg/ml) and addition (100 μg/ml)] for 24 h. IL-12p40 mRNA expression was determined by RT-qPCR. Data are presented as mean ± SD (*n* ≥ 3).

To investigate whether exosomal nucleic acids are sufficient for the NKG2D ligand expression, exosomal RNA and DNA were extracted from exosomes released from HepG2 cells with pHBV, and THP-1 macrophages were stimulated with exosomal nucleic acids. The expression of ULBP2 in THP-1 macrophages was substantially increased by stimulation with exosomal RNA and DNA (Figure 5A). Viral RNA and DNA are well known to activate MyD88, TICAM-1, and VISA pathways, and thus, we investigated whether stimulation of TLRs and RLRs pathway mimics EVs that were released from HBV-infected cells. To stimulates the TLR3–TICAM-1 pathway, 50 μg of polyI:C was added to culture medium, and 1 μg of polyI:C was transfected to stimulate the RLRs–VISA pathway. For stimulation of TLR7 and TLR9, which trigger the signal *via* MyD88, 1 μg/ml of CL097 and 500 μM of ODN2216 were added to cell culture medium, respectively. The simultaneous stimulation of all four pathways with TLR3/RIG-I/MDA5, TLR7, and TLR9 ligands (polyI:C, CL097, and ODN2216) induced the expression of IL-12p35 as well as ULBP1 and ULBP2, whereas the stimulation of each separate pathway failed to increase the expression levels (Figure 5B and Figures S3A,B in Supplementary Material). IL12-p40 mRNA expression was increased by stimulation with both polyI:C (addition and transfection) and CL097, even in the absence of ODN2216 (Figure S3B in Supplementary Material). Given that EVs released by hepatocytes with pHBV did not increase IL-12p35 mRNA expression in THP-1 macrophages (Figure 3F), we hypothesized that EVs contain the agonists for each pathway as well as other molecules that regulate innate immune response.

The exosomes deliver miR to other cells and regulate the innate immune response (11). Thus, we investigated the expression levels of 17 immunoregulatory miRs within EVs and exosomes (23). Interestingly, HBV increased the expression levels of miR-21 and miR-29a in EVs and CD81^+^ exosomes (Figure 5C and Figure S3C in Supplementary Material). Moreover, exosomal miR-21 and miR-29a levels were markedly increased by infection of HepG2-NTCP cells with HBV (Figure 5D). miR-21 downregulates IL-12p35 mRNA expression (24) and is induced by the HBx proteins (25). miR-29a is known to suppress IL-12p40 mRNA expression (26).

Interestingly, EVs released from hepatocytes reduced IL-12p40, but not IFN-β expression, in THP-1 macrophages that were stimulated with CL097 and polyI:C (addition and transfection) (Figure 5E). Moreover, EVs released from HepG2 with pHBV reduced stimulation-induced IL-12p35 expression in THP-1 macrophages compared to EVs from HepG2 without pHBV (Figure 5F). We confirmed that the expression of HBV in hepatocytes increased the levels of miR-21, miR-29a, and other immunoregulatory miRs in THP-1 macrophages *via* EVs (Figure 5G). In addition, miR-29a transfection to THP-1 macrophage decreased IL-12p40 expression (Figure S3D in Supplementary Material), and EVs released from hepatocytes that were transfected with miR-29a decreased IL-12p40 expression in THP-1 macrophages (Figure 5H). Overall, these results indicate that HBV-induced immunosuppressive miRs were transferred to macrophages from hepatocytes, thereby suppressing the IL-12 expression.

Hepatitis B virus intravenous infection increased the expression of a NKG2D ligand, ULBP3, in tree shrew liver but failed to increase IL-12p35 and IL-12p40 *in vivo* (Figure 1D). These data are nicely correlated with our *in vitro* studies.

### IFN-γ Promotes Viral RNA Degradation in Hepatocytes

**Figure 6:**
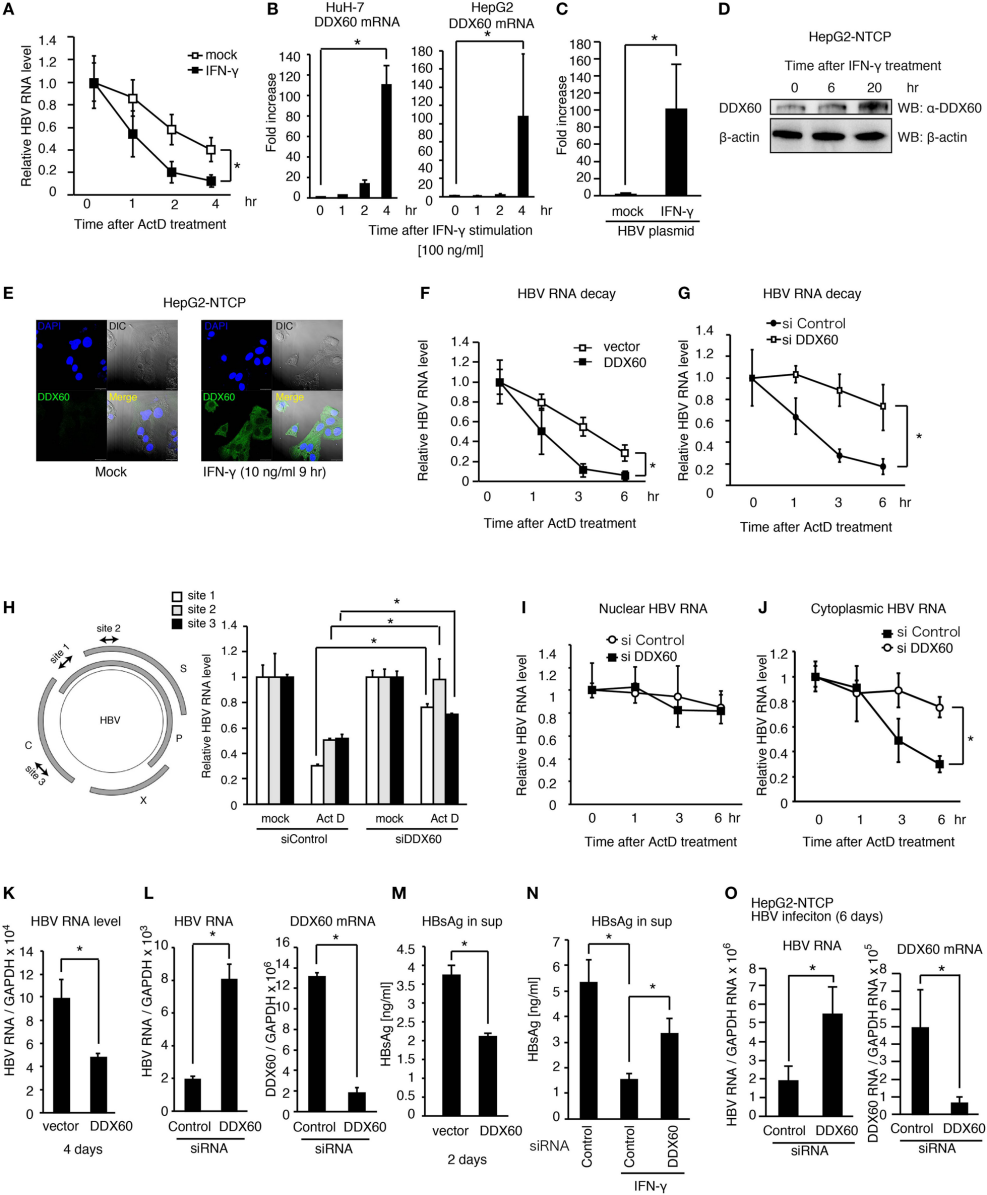
**DDX60 promotes cytoplasmic HBV RNA degradation**. **(A)** HuH-7 cells transfected with pHBV were treated with 10 ng/ml of IFN-γ for 1 day. Cells were treated with actinomycin D (ActD), and then HBV RNA degradation was determined by RT-qPCR and normalized to GAPDH. **(B)** HuH-7 and HepG2 cells were stimulated with 10 ng/ml of IFN-γ. Total RNA was extracted at indicated time points, and mRNA levels were determined by RT-qPCR and normalized to GAPDH. Data are presented as mean ± SD (*n* = 3). **(C)** HepG2-T23 cells were transfected with pHBV for 24 h. Cells were stimulated with 10 ng/ml of IFN-γ, and the expression of DDX60 was determined by RT-qPCR. Data are presented as mean ± SD (*n* = 3). **(D)** HepG2-NTCP cells were stimulated with 10 ng/ml of IFN-γ, and whole cell extract was prepared at indicated time points. The proteins were subjected to SDS-PAGE and were detected by western blotting with anti-DDX60 and anti-β actin antibodies. **(E)** HepG2-NTCP cells were fixed and labeled with anti-DDX60 antibody. Cells were stained with DAPI and Alexa Fluor-488 secondary antibody and were observed using confocal microscopy. The scale bar represents 10 μm. **(F–J)** HuH-7 cells transfected with pHBV together with an empty vector or a DDX60 expression vector **(F)** or siRNA for control or DDX60 **(G–J)** were treated with ActD. Total RNA **(F–H)**, nuclear RNA **(I)**, and cytoplasmic RNA **(J)** were extracted at indicated time points. HBV RNA levels were determined using RT-qPCR and normalized to GAPDH (*n* = 3). **(K–M)** HuH-7 cells transfected with the combination of pHBV and either an empty vector or a DDX60 expression vector **(K,M)** or with pHBV and an siRNA (as a negative control) or DDX60 **(L)**. Four days after transfection, total RNA was extracted, and HBV RNA and DDX60 mRNA levels were determined by RT-qPCR and normalized to GAPDH **(K,L)**. HBsAg levels in the culture medium 2 days after transfection were determined using ELISA **(M)**. **(N)** siRNA for control or DDX60 was transfected into HuH-7 cells with the HBV plasmid. Two days after transfection, cells were washed with fresh medium and subsequently treated with or without 10 ng/ml IFN-γ for 2 days. HBsAg levels in culture medium were determined by ELISA. **(O)** HepG2-NTCP cells were infected with infectious HBV particles for 6 days. Total RNA was extracted from the HBV-infected cells. HBV RNA and DDX60 mRNA levels were determined using RT-qPCR and normalized to GAPDH. Data are presented as mean ± SD (*n* ≥ 3).

IFN-γ itself has many functions, such as promotion of Th1 differentiation and macrophage activation. A previous study showed that IFN-γ destabilizes viral RNA (15); however, underlying mechanism is not fully elucidated. To investigate the physiological meaning of EVs-induced IFN-γ production in antiviral innate immune response, we sought to assess the role of IFN-γ in viral RNA degradation. To observe viral RNA degradation, HepG2 cells were transfected with pHBV, and viral RNA expression was attenuated by actinomycin D (ActD), and then viral RNA levels were determined by RT-qPCR. As previously reported, IFN-γ treatment promoted the degradation of viral RNA (Figure 6A). Interestingly, IFN-γ treatment increased the DDX60 mRNA and protein levels in hepatocytes (Figures 6B–E). It has been shown that DDX60 is involved in a viral RNA degradation pathway (6). The ectopic expression of DDX60 promoted the degradation of HBV RNA (Figure 6F). In contrast, DDX60 knockdown delayed the degradation of HBV RNA (Figures 6G,H). Interestingly, cytoplasmic viral RNA was degraded faster than nuclear viral RNA, and DDX60 knockdown delayed the degradation of cytoplasmic viral RNA but not nuclear viral RNA (Figures 6I,J). These data suggest that IFN-γ induces the expression of DDX60, which promotes viral RNA degradation.

Next, we assessed the role of DDX60 in viral replication. The ectopic expression of DDX60 decreased the HBV RNA levels in hepatocytes with pHBV (Figure 6K), and DDX60 knockdown substantially increased HBV RNA levels (Figure 6L). The HBsAg levels were also reduced in the culture medium by DDX60 ectopic expression (Figure 6M). IFN-γ-treatment reduced HBsAg, and the IFN-γ-mediated reduction of HBsAg was alleviated by DDX60 knockdown (Figure 6N), thereby suggesting the importance of DDX60 in the IFN-γ-antiviral activity against HBV.

Next, we further assessed the role of DDX60 during viral infection. IFN-γ treatment reduced the viral RNA levels in HBV-infected HePG2-NTCP cells (Figure S4A in Supplementary Material), and *DDX60* knockdown increased the HBV RNA levels in cells infected with HBV for 6 days (Figure 6O). Collectively, these results indicate that DDX60-mediated HBV RNA degradation suppresses viral replication. Using a hydrodynamic injection mouse model, we confirmed that IFN-γ treatment could reduce the serum HBsAg level *in vivo* after HBV injection into mouse liver (Figure S4B in Supplementary Material). These results support a model that exosomes-mediated hepatic IFN-γ production plays a crucial role in antiviral innate immune response to HBV.

## Discussion

In the present study, we elucidated the early *in vivo* innate immune response to HBV infection in tree shrews. IFN-γ was induced in the liver at 1 day post-infection, and this early production of IFN-γ suggests the importance of hepatic group 1 ILCs for the *in vivo* innate immune response. Experimental tree shrew infection with HBV was successful in approximately 55% of the animals inoculated, whereas the remaining animals rejected HBV (27), suggesting that the antiviral innate immune response in tree shrews has the ability to eliminate HBV. The major group 1 ILCs in liver is NK cells, and we determined that hepatic NK cells were activated in the presence of hepatic F4/80^+^ cells. This is consistent with the upregulation of CD69, an NK cell activation marker, in tree shrew liver after HBV infection. However, we do not exclude the possibility that not only NK cells but also other types of cells produced IFN-γ. In woodchuck animal model, woodchucks infected with closely related woodchuck hepatitis virus also showed the upregulation of NKp46, an NK cell activating receptor, immediately after infection (28). Chisari and colleagues first implied the importance of NK cell influx for early non-cytopathic control of HBV based on a study using a chimpanzee animal model (29). Accumulating evidence indicates that NK cells are major determinants of the clinical outcome following infection with HBV (30), and NK cells have been reported to control HBV infection in humans (31, 32). Considering these observations, we prefer the interpretation that HBV infection activates hepatic NK cells, resulting in the early IFN-γ production in the liver. Although hepatic IFN-γ expression levels increased only 20-fold after infection of tree shrews with HBV, non-parenchymal cells would produce much more amount of IFN-γ after HBV infection, because parenchymal cells do not produce IFN-γ.

There are several reports indicating that IFN-γ exhibits antiviral activity against HBV. For example, knockout of IFN-γ increased HBV replication in mouse liver (33), and IFN-γ expression inhibited HBV replication *in vitro* (34). Previously, Chisari and colleagues showed that IFN-γ and TNF-α destabilize viral RNA and abolish HBV gene expression and replication in the liver without killing hepatocytes (15). Here, we demonstrated that IFN-γ induces DDX60 expression in hepatocytes, leading to DDX60-mediated HBV RNA degradation. We also showed that DDX60 deficiency leads to increased HBV RNA levels. These findings reveal the molecular mechanism underlying the IFN-γ-mediated destabilization of viral RNA. Recently, Protzer and colleagues reported that TNF-α and IFN-γ induce the deamination of covalently closed circular HBV DNA, resulting in HBV DNA instability (35). These observations suggest that IFN-γ together with TNF-α destabilizes viral RNA and DNA. A previous study showed that ZAP(S) is involved in nuclear HBV RNA degradation *via* the ribonuclease complex (36), whereas we showed that DDX60 is involved in not nuclear but cytoplasmic viral RNA degradation. These observations indicate that DDX60 has a distinct role in viral RNA degradation.

Extracellular vesicles, including exosomes, have important functions in intercellular communication (37), and exosomes deliver HCV RNA to dendritic cells, thereby inducing the innate immune response (38). Here, we demonstrated that EVs released from HBV-infected hepatocytes contained viral nucleic acids, and exosomal viral nucleic acids stimulated macrophages. These observations indicate that EVs including exosomes play a crucial role in the innate immune response against HBV.

Several viruses have evolved to escape the host innate immune response. Recent studies have revealed the molecular mechanism underlying viral escape from the host innate immune response (39). It has been shown that HBV increases miR-21 expression in hepatocytes (25). Here, we showed that exosomal miR-21 as well as other immunosuppressive miRs were increased by the HBV infection of hepatocytes, and these exosomal miRs downregulated IL-12 expression, which is a cytokine well known to activate NK cells. In another animal model, woodchuck hepatitis virus infection led to only a temporary expression of IL-12 at 3–6 h post-infection in woodchuck liver, and IL-12 expression was not detected 18 h after infection or at any later time points up to 2 weeks (28). In addition, in chronic hepatitis B patients, NK cell function has been reported to be attenuated by HBV infection (40). Exosome-mediated IL-12 downregulation would be a mechanism of viral escape from the host innate immune response. Recently, it has been shown that serum exosomes isolated from patients chronically infected with HBV regulates NK cell function (41). This suggests that exosomes regulate the innate immune system during not only the early phase but also the chronic phase of viral infection. The balance between the host innate immune response and virus-mediated suppression might determine whether HBV persistently infects the liver.

Overall, our results provide novel insights into the molecular mechanisms underlying EVs-mediated innate immune response during viral infection.

## Experimental Procedures

### Animals

Tree shrews (*T. belangeri chinensis*) were injected intravenously with 10^7^ copies of infectious HBV particles (C_JPNAT, accession number: AB246345.1). Tissues were extracted at days 0, 1, and 3. Total RNA was extracted with TRIzol (Invitrogen). C57BL/6 and BALB/c mice were purchased from Hokudo and SANKYO LABO SERVICE. All of the animal studies were conducted in strict accordance with the Guidelines for Animal Experimentation of the Japanese Associations for Laboratory Animal Science. The protocols were approved by the Animal Care and Use Committee of Hokkaido University, Japan (permit numbers: 09-0215, 13-0049, and 15-0017) and the ethics committee of the Tokyo Metropolitan Institute of Medical Science.

### Cells, Virus, and Reagents

C57BL/6 mouse liver was dissociated into single-cell suspensions using a Liver Dissociation Kit (Miltenyi Biotec), according to the manufacturer’s instructions. Hepatic F4/80^+^ cells were isolated using anti-F4/80-biotin antibody and anti-biotin MicroBeads (Miltenyi Biotec). Liver NK cells were isolated using an NK cell isolation kit II (Miltenyi Biotec), according to the manufacturer’s instruction. THP-1 cells were purchased from JCRB cell bank and cultured in RPMI-1640 with 5% FCS. THP-1 cells were differentiated into macrophages with 60 ng/ml of PMA for 16 h. For stimulation of THP-1 macrophages, ODN2216 (500 μM), CL097 (1 μg/ml), and polyI:C (50 μg/ml) were added to cell culture, or polyI:C (50 μg/ml) were transfected into cells using 2 μl of lipofectamine 2000 (addition and transfection) in 24-well plate. For activating VISA pathway, 1 μg of polyI:C were transfected into cells in 24-well plate. HepG2-NTCP cells were cultured with D-MEM/F-12 + GlutaMax with 10 mM HEPES, 200 U/ml penicillin, 200 μg/ml streptomycin, 10% FCS, 50 μM hydrocortisone, and 5 μg/ml insulin. HepG2-T23 cells, which stably express HBV, were kindly donated by Chayama (Hiroshima University) (42). HepG2-NTCP cells, which stably express NTCP for the HBV receptor, were donated by Watashi (NIID) (43). Human primary hepatocytes and human primary hepatic stellate cells were purchased from ScienCell Research Laboratories. We used human and mouse IFN-γ (Cell Signaling Technology), actinomycin D (Life Technologies), anti-DDX60 antibody (SIGMA-Aldrich), anti-β-actin antibody (AC-15: SIGMA-Aldrich), and an HBsAg ELISA kit (XpressBio). ExoAB antibody kit, which includes anti-CD9, anti-CD63, and anti-CD81 antibodies, was purchased from System Biosciences.

### Confocal Microscopy

HepG2-NTCP cells were treated with 10 ng/ml of IFN-γ for 8 h. Cells were washed with PBS, fixed with formaldehyde, and permeabilized with 0.2% of Triton-X 100 in PBS. Cells were subsequently blocked with 1% of BSA in PBS for 10 min and then incubated with anti-DDX60 rabbit polyclonal antibody (SIGMA-Aldrich) at 1/100 dilution. Cells were washed 4× with 1% of BSA in PBS and stained with Alexa Fluor 488 anti-rabbit antibodies. Cells were embedded with ProLong Gold Antifade Mountant with DAPI (Life Technologies).

### RNA-Seq

Total RNA extracted from tissues of tree shrew was extracted using TRIZOL (Invitrogen), according to the manufacturer’s instruction. RNA-seq libraries were prepared using TruSeq RNA-seq kit (illumina). Sequencing libraries were sequenced on MiSeq using MiSeq sequencing reagent kit ver2 (illumina), according to the manufacturer’s instruction. The RNA-seq tags were then mapped to the reference transcripts of *Tupaia chinensis*. Normalization and transformation of expression values and hierarchal clustering analysis were carried out using CLC genomics workbench software. RNA-seq data have been deposited to DRA in DDBJ (accession number: DRA004164).

### ELISA

NK and F4/80^+^ cells were isolated from mouse liver. The 1 × 10^6^ NK cells and 1 × 10^6^ F4/80^+^ cells were co-cultured with 2 × 10^5^ HepG2 cells expressing HBV for 24 h. Culture supernatant was collected and analyzed for mouse IFN-γ by ELISA (GE Healthcare). Concentration of HBV surface antigen (HBsAg) in the serum and culture supernatant was quantified by ELISA, according to the manufacturer’s instruction (XpressBio).

### Hydrodynamic Injection

Hydrodynamic injection was performed with TransIT-EE Hydrodynamic Delivery Solution (Takara), as described previously (44).

### Isolation of Exosomes

HepG2 or HuH-7 cells cultured in 60-mm dish were washed twice with serum-free medium and further incubated with serum-free medium for 24 h. Culture supernatant was recovered and centrifuged at 2000 rpm for 30 min to remove debris. Obtained supernatant was mixed with total exosome isolation solution and incubated at 4°C for 24 h, according to the manufacturer’s instruction (Thermo Fisher Scientific). Mixture was centrifuged at 1000 × *g* for 1 h, and then pellet was suspended with 0.5 ml of serum-free medium. The 0.1 ml of suspended exosome solution was added to 1 well of 24-well plate to activate THP-1 cells. CD81^+^ exosomes were isolated using cell culture total exosome isolation kit and CD81^+^ exosome isolation kit, according to the manufacturer’s instruction (Thermo Fisher Scientific).

### HBV RNA Decay

The plasmid carrying the 1.4× HBV genome (HBV plasmid), in which HBV RNA is transcribed from the pCMV minimal promoter, was kindly gifted by Chayama. The DDX60 expression vector has been described elsewhere (6). HuH-7 cells were transfected with 0.2 μg of HBV plasmid using Lipofectamine 2000 reagent (Invitrogen). One to 3 days after transfection, the cells were treated with 10 μg/ml actinomycin D in order to inhibit HBV RNA transcription. Total RNA was extracted with the TRIZOL reagent (Invitrogen), according to the manufacturer’s instructions. Cytoplasmic and nuclear RNA were extracted with the Cytoplasmic and Nuclear RNA purification kit (NORGEN BIOTEK CORP.), according to the manufacturer’s instructions. Viral RNA levels were determined by qPCR.

### qPCR

Reverse transcription reactions of mRNA and miR were performed using the High-Capacity cDNA Reverse Transcription Kit (Life Technologies) and Mir-X miRNA First-strand Synthesis kit (Takara). Real-time PCR was performed with the SYBR Green Real-Time PCR Master Mix (Life Technologies) using the Step One Real-Time PCR System (Life Technologies). PCR primers used for the RT-qPCR procedures are described in the Supplementary Material.

### HBV Infection

To determine exosomal miR levels, HepG2-NTCP cells were seeded onto 24-well plate. The 40,000 genome copies of HBV isolated from HepG2-T23 cell culture medium were infected to HepG2-NTCP cells for 3 days. Cells were washed 3× with cell culture medium and were further incubated for 3 days. CD81^+^ exosomes were isolated from cell culture medium, and exosomal miR and HBV RNA levels were determined by RT-qPCR and normalized to U6 level.

To investigate the role of DDX60 in the suppression of viral replication, HBV particles isolated from the cell culture supernatants of HepG2-T23 cells were used to infect HepG2-NTCP cells at 40,000 genome copies per well in a 24-well plate.

To determine the ULBP1 and ULBP2 mRNA levels in THP-1 macrophages co-cultured with HBV-infected HepG2-NTCP cells, HepG2-NTCP cells were infected with 400,000 genome copies of HBV for 9 days and subsequently co-cultured with THP-1 macrophages using transwell co-culture system (pore size 3 μm) for 2 days. Total RNA of THP-1 macrophages were isolated with TRIZOL.

Primary human hepatocytes and human hepatic stellate cells were infected with 1,000,000 genome copies of HBV for 24 h in 24-well plates.

## Author Contributions

TaS, NY, KT-K, MK, and HO conducted and/or supervised tree shrew analyses; CL developed the method for HBV *in vitro* studies; and TK, YF, TD, EM, and HO conducted *in vitro* studies. TK, YF, and HO conducted the research of exosomes, and HO performed the experiments related to viral RNA degradation. TsS, MM, and HO supervised the study, and HO wrote the manuscript.

## Conflict of Interest Statement

The authors declare that the research was conducted in the absence of any commercial or financial relationships that could be construed as a potential conflict of interest.

## References

[1] Kawai T Akira S. Toll-like receptors and their crosstalk with other innate receptors in infection and immunity. Immunity (2011) 34:637–50. 10.1016/j.immuni.2011.05.006 21616434

[2] Loo YM Gale M Jr. Immune signaling by RIG-I-like receptors. Immunity (2011) 34:680–92. 10.1016/j.immuni.2011.05.003 21616437 PMC3177755

[3] Li XD Wu J Gao D Wang H Sun L Chen ZJ. Pivotal roles of cGAS-cGAMP signaling in antiviral defense and immune adjuvant effects. Science (2013) 341:1390–4. 10.1126/science.1244040 23989956 PMC3863637

[4] Schoggins JW MacDuff DA Imanaka N Gainey MD Shrestha B Eitson JL Pan-viral specificity of IFN-induced genes reveals new roles for cGAS in innate immunity. Nature (2014) 505:691–5. 10.1038/nature12862 24284630 PMC4077721

[5] Unterholzner L Keating SE Baran M Horan KA Jensen SB Sharma S IFI16 is an innate immune sensor for intracellular DNA. Nat Immunol (2010) 11:997–1004. 10.1038/ni.1932 20890285 PMC3142795

[6] Oshiumi H Miyashita M Okamoto M Morioka Y Okabe M Matsumoto M DDX60 is involved in RIG-I-dependent and independent antiviral responses, and its function is attenuated by virus-induced EGFR activation. Cell Rep (2015) 11:1193–207. 10.1016/j.celrep.2015.04.047 25981042

[7] Oshiumi H Mifsud EJ Daito T. Links between recognition and degradation of cytoplasmic viral RNA in innate immune response. Rev Med Virol (2016) 26:90–101. 10.1002/rmv.1865 26643446

[8] Simons M Raposo G Exosomes – vesicular carriers for intercellular communication. Curr Opin Cell Biol (2009) 21:575–81. 10.1016/j.ceb.2009.03.007 19442504

[9] Dreux M Garaigorta U Boyd B Decembre E Chung J Whitten-Bauer C Short-range exosomal transfer of viral RNA from infected cells to plasmacytoid dendritic cells triggers innate immunity. Cell Host Microbe (2012) 12:558–70. 10.1016/j.chom.2012.08.010 23084922 PMC3479672

[10] Okamoto M Oshiumi H Azuma M Kato N Matsumoto M Seya T. IPS-1 is essential for type III IFN production by hepatocytes and dendritic cells in response to hepatitis C virus infection. J Immunol (2014) 192:2770–7. 10.4049/jimmunol.1301459 24532585

[11] Alexander M Hu R Runtsch MC Kagele DA Mosbruger TL Tolmachova T Exosome-delivered microRNAs modulate the inflammatory response to endotoxin. Nat Commun (2015) 6:7321. 10.1038/ncomms8321 26084661 PMC4557301

[12] Hulsmans M Holvoet P. MicroRNA-containing microvesicles regulating inflammation in association with atherosclerotic disease. Cardiovasc Res (2013) 100:7–18. 10.1093/cvr/cvt161 23774505

[13] Sato S Li K Kameyama T Hayashi T Ishida Y Murakami S The RNA sensor RIG-I dually functions as an innate sensor and direct antiviral factor for hepatitis B virus. Immunity (2015) 42:123–32. 10.1016/j.immuni.2014.12.016 25557055

[14] Dansako H Ueda Y Okumura N Satoh S Sugiyama M Mizokami M The cyclic GMP-AMP synthetase-STING signaling pathway is required for both the innate immune response against HBV and the suppression of HBV assembly. FEBS J (2016) 283:144–56. 10.1111/febs.13563 26471009

[15] Guidotti LG Ishikawa T Hobbs MV Matzke B Schreiber R Chisari FV. Intracellular inactivation of the hepatitis B virus by cytotoxic T lymphocytes. Immunity (1996) 4:25–36. 10.1016/S1074-7613(00)80295-2 8574849

[16] Wieland S Thimme R Purcell RH Chisari FV. Genomic analysis of the host response to hepatitis B virus infection. Proc Natl Acad Sci U S A (2004) 101:6669–74. 10.1073/pnas.0401771101 15100412 PMC404103

[17] Tsukiyama-Kohara K Kohara M. *Tupaia belangeri* as an experimental animal model for viral infection. Exp Anim (2014) 63:367–74. 10.1538/expanim.14-0007 25048261 PMC4244285

[18] Carloni V Mazzocca A Ravichandran KS. Tetraspanin CD81 is linked to ERK/MAPKinase signaling by Shc in liver tumor cells. Oncogene (2004) 23:1566–74. 10.1038/sj.onc.1207287 14676841

[19] Spits H Artis D Colonna M Diefenbach A Di Santo JP Eberl G Innate lymphoid cells – a proposal for uniform nomenclature. Nat Rev Immunol (2013) 13:145–9. 10.1038/nri3365 23348417

[20] Boltjes A Movita D Boonstra A Woltman AM. The role of Kupffer cells in hepatitis B and hepatitis C virus infections. J Hepatol (2014) 61:660–71. 10.1016/j.jhep.2014.04.026 24798624

[21] Ebihara T Masuda H Akazawa T Shingai M Kikuta H Ariga T Induction of NKG2D ligands on human dendritic cells by TLR ligand stimulation and RNA virus infection. Int Immunol (2007) 19:1145–55. 10.1093/intimm/dxm073 17878262

[22] Kloss M Decker P Baltz KM Baessler T Jung G Rammensee HG Interaction of monocytes with NK cells upon Toll-like receptor-induced expression of the NKG2D ligand MICA. J Immunol (2008) 181:6711–9. 10.4049/jimmunol.181.10.6711 18981088

[23] Smyth LA Boardman DA Tung SL Lechler R Lombardi G. MicroRNAs affect dendritic cell function and phenotype. Immunology (2015) 144:197–205. 10.1111/imm.12390 25244106 PMC4298414

[24] Lu TX Munitz A Rothenberg ME. MicroRNA-21 is up-regulated in allergic airway inflammation and regulates IL-12p35 expression. J Immunol (2009) 182:4994–5002. 10.4049/jimmunol.0803560 19342679 PMC4280862

[25] Qiu X Dong S Qiao F Lu S Song Y Lao Y HBx-mediated miR-21 upregulation represses tumor-suppressor function of PDCD4 in hepatocellular carcinoma. Oncogene (2013) 32:3296–305. 10.1038/onc.2013.150 23604124

[26] Brain O Owens BM Pichulik T Allan P Khatamzas E Leslie A The intracellular sensor NOD2 induces microRNA-29 expression in human dendritic cells to limit IL-23 release. Immunity (2013) 39:521–36. 10.1016/j.immuni.2013.08.035 24054330

[27] Yan RQ Su JJ Huang DR Gan YC Yang C Huang GH Human hepatitis B virus and hepatocellular carcinoma. I. Experimental infection of tree shrews with hepatitis B virus. J Cancer Res Clin Oncol (1996) 122:283–8. 10.1007/BF01261404 8609151 PMC12201021

[28] Guy CS Mulrooney-Cousins PM Churchill ND Michalak TI. Intrahepatic expression of genes affiliated with innate and adaptive immune responses immediately after invasion and during acute infection with woodchuck hepadnavirus. J Virol (2008) 82:8579–91. 10.1128/JVI.01022-08 18596101 PMC2519695

[29] Guidotti LG Rochford R Chung J Shapiro M Purcell R Chisari FV. Viral clearance without destruction of infected cells during acute HBV infection. Science (1999) 284:825–9. 10.1126/science.284.5415.825 10221919

[30] Busca A Kumar A. Innate immune responses in hepatitis B virus (HBV) infection. Virol J (2014) 11:22. 10.1186/1743-422X-11-22 24507433 PMC3922976

[31] Fisicaro P Valdatta C Boni C Massari M Mori C Zerbini A Early kinetics of innate and adaptive immune responses during hepatitis B virus infection. Gut (2009) 58:974–82. 10.1136/gut.2008.163600 19201769

[32] Bertoletti A Ferrari C. Innate and adaptive immune responses in chronic hepatitis B virus infections: towards restoration of immune control of viral infection. Gut (2012) 61:1754–64. 10.1136/gutjnl-2011-301073 22157327

[33] McClary H Koch R Chisari FV Guidotti LG. Relative sensitivity of hepatitis B virus and other hepatotropic viruses to the antiviral effects of cytokines. J Virol (2000) 74:2255–64. 10.1128/JVI.74.5.2255-2264.2000 10666256 PMC111707

[34] Kan QC Li DL Yu ZJ. Vector-mediated expression of interferon gamma inhibits replication of hepatitis B virus in vitro. Acta Virol (2013) 57:421–8. 10.4149/av_2013_04_421 24294955

[35] Xia Y Stadler D Lucifora J Reisinger F Webb D Hosel M Interferon-gamma and tumor necrosis factor-alpha produced by T cells reduce the HBV persistence form, cccDNA, without cytolysis. Gastroenterology (2016) 150:194–205. 10.1053/j.gastro.2015.09.026 26416327

[36] Mao R Nie H Cai D Zhang J Liu H Yan R Inhibition of hepatitis B virus replication by the host zinc finger antiviral protein. PLoS Pathog (2013) 9:e1003494. 10.1371/journal.ppat.1003494 23853601 PMC3708887

[37] Schorey JS Harding CV. Extracellular vesicles and infectious diseases: new complexity to an old story. J Clin Invest (2016) 126:1181–9. 10.1172/JCI81132 27035809 PMC4811125

[38] Robbins PD Morelli AE. Regulation of immune responses by extracellular vesicles. Nat Rev Immunol (2014) 14:195–208. 10.1038/nri3622 24566916 PMC4350779

[39] Chan YK Gack MU. Viral evasion of intracellular DNA and RNA sensing. Nat Rev Microbiol (2016) 14:360–73. 10.1038/nrmicro.2016.45 27174148 PMC5072394

[40] Maini MK Peppa D. NK cells: a double-edged sword in chronic hepatitis B virus infection. Front Immunol (2013) 4:57. 10.3389/fimmu.2013.00057 23459859 PMC3585438

[41] Yang Y Han Q Hou Z Zhang C Tian Z Zhang J. Exosomes mediate hepatitis B virus (HBV) transmission and NK-cell dysfunction. Cell Mol Immunol (2016). 10.1038/cmi.2016.24 27238466 PMC5423088

[42] Hayes CN Akamatsu S Tsuge M Miki D Akiyama R Abe H Hepatitis B virus-specific miRNAs and Argonaute2 play a role in the viral life cycle. PLoS One (2012) 7:e47490. 10.1371/journal.pone.0047490 23091627 PMC3472984

[43] Iwamoto M Watashi K Tsukuda S Aly HH Fukasawa M Fujimoto A Evaluation and identification of hepatitis B virus entry inhibitors using HepG2 cells overexpressing a membrane transporter NTCP. Biochem Biophys Res Commun (2014) 443:808–13. 10.1016/j.bbrc.2013.12.052 24342612

[44] Leong CR Oshiumi H Okamoto M Azuma M Takaki H Matsumoto M A MAVS/TICAM-1-independent interferon-inducing pathway contributes to regulation of hepatitis B virus replication in the mouse hydrodynamic injection model. J Innate Immun (2015) 7:47–58. 10.1159/000365113 25115498 PMC6951042

