# Supplementary material for: Extracellular Vesicles Including Exosomes Regulate Innate Immune Responses to Hepatitis B Virus Infection
Source: Front Immunol. 2016 Aug 31;7:335. doi: 10.3389/fimmu.2016.00335 (PMC5005343; doi:10.3389/fimmu.2016.00335)

Supplemental Information

Primer list used for qPCR

Tree shrew IFI16-qF:	AATGTTCCATGCCACAGTGG
Tree shrew IFI16-qR:	AGAGATTGTATATCTCCAGG
Tree shrew cGAS-qF:	TCACACATGTACCGAGAACC
Tree shrew cGAS-qR:	GAGAATAGATTAACTCCAGG
Tree shrew IFN-b-qF:	CTTCGATTCCAACTAAGTGG
Tree shrew IFN-b-qR:	CCTCGAAGCTCATACGGTCC
Tree shrew IFN-l3-qF:	GAAGAGTCGCTCTTGCAGAAGG
Tree shrew IFN-l3-qR:	GTGGATGTGGCGTAGCACGTGC
Tree shrew DDX60-qF:	TTATCACATGTGTATGTGCG
Tree shrew DDX60-qR:	ACTACGGCAAATTGTCCTCC
Tree shrew RIG-I-qF:	CATGCTACTATACTTCTCTAGG
Tree shrew RIG-I-qR:	TGAAAATACCACTGAAATAGCC
Tree shrew IFN-g-qF:	GGTCATCAAAAAGAGTGTGG
Tree shrew IFN-g-qR:	GGTCAATCATCACATTGAGG
Tree shrew b-actin qF:	ATGTGGACATCCGTAAGGAC
Tree shrew b-actin qR:	CAGGATGGAGCCACCAATCC
Tree shrew IL-12p35 qF:	CCATAAATGCCCAGCTTTGG
Tree shrew IL-12p35 qR:	CACGCACTCTGAAAGCGTGG
Tree shrew IL-12p40 qF:	AGTGAAATAGTAGGATCTGG
Tree shrew IL-12p40 qR:	CCTTAAAATATCAGTGGACC
Tree shrew UBLP3 qF:	GGAAGATCAGGTGATAACGC
Tree shrew ULBP3 qR:	AAGAGGAGGAACATCTGTCC
HBV-C hiroshima qF:	ATGCCTGCTAGGTTTTATCC
HBV-C tranomon qF (site 1):	ATGCCTGCTAGGTTCTATCC
HBV-C tranomon qR (site 1):	TTCCCAAGAATATGGTGACC
HBV-C tranomon qF2 (site 2):	ACAACATCAGGATTCCTAGG
HBV-C tranomon qR2 (site 2):	TAGCCAGGACAAATTGGAGG
HBV-C tranomon qF4 (site 3):	GGCAAGCTATTCTGTGTTGG
HBV-C tranomon qR4 (site 3):	TCCACACTCCAAAAGACACC
Human GAPDH qF:	GAGTCAACGGATTTGGTCGT

Human GAPDH qR:	TTGATTTTGGAGGGATCTCG
Human IFN-b qF:	TGGGAGGATTCTGCATTACC
Human IFN-b qR:	CAGCATCTGCTGGTTGAAGA
Human IFN-g qF:	ACTGACTTGAATGTCCAACGCA
Human IFN-g qR:	ATCTGACTCCTTTTTCGCTTCC
Human IFN-l1 qF:	CGCCTTGGAAGAGTCACTCA
Human IFN-l1 qR:	GAAGCCTCAGGTCCCAATTC
let-7i	TGAGGTAGTAGTTTGTGCTGTT
miR-21	TAGCTTATCAGACTGATGTTGA
miR-22	AAGCTGCCAGTTGAAGAACTGT
miR-23b	ATCACATTGCCAGGGATTACC
miR-29c	TAGCACCATTTGAAATCGGTTA
miR-29b	TAGCACCATTTGAAATCAGTGTT
miR-29a	TAGCACCATCTGAAATCGGTTA
miR-30b	TGTAAACATCCTACACTCAGCT
miR-34a	TGGCAGTGTCTTAGCTGGTTGT
miR-99a	AACCCGTAGATCCGATCTTGTG
miR-107	AGCAGCATTGTACAGGGCTATCA
miR-125a	TCCCTGAGACCCTTTAACCTGTGA
miR-126	TCGTACCGTGAGTAATAATGCG
miR-142-3p	TGTAGTGTTTCCTACTTTATGGA
miR-142	CATAAAGTAGAAAGCACTACT
miR-146a	TGAGAACTGAATTCCATGGGTT
miR-148b	TCAGTGCATCACAGAACTTTGT
miR-148a	TCAGTGCACTACAGAACTTTGT
miR-152	AGGTTCTGTGATACACTCCGACT
miR-155	TTAATGCTAATCGTGATAGGGGT
miR-221	AGCTACATTGTCTGCTGGGTTTC
miR-301a	GCTCTGACTTTATTGCACTACT
miR-451	AAACCGTTACCATTACTGAGTT

Supplemental Documents

Supplemental Figure S1

(A, B) Box (A) and dot (B) plots of gene expression in the liver at 0, 1, and 3 days post.

(C) Expression values of genes involved in antiviral innate immune response.

(D) The gene expression levels in the spleen and kidney of HBV-infected tree shrews were determined using RT-qPCR and was normalized against that of β-actin.

(E) RT-qPCR analysis of HBV RNA, IFN-β, IFN-γ, IFN-λ1, and DDX60 in HepG2 cells transfected with HBV plasmids.

Supplemental Figure S2

(A) $CD81^+$ exosomes were isolated from EVs released by HepG2 transfected with mock or pHBV for 24 hr. DNA was extracted from exosomes, and HBV DNA was detected by RT-PCR with HBV tranomon-C qF and qR primers. PCR product was subjected to agarose gel electrophoresis and stained with etidium bromide.

(B) HepG2-NTCP cells were infected with HBV for six days. RNA was extracted from $CD81^+$ exosomes released from HBV-infected HepG2-NTCP cells, and HBV RNA level was determined by RT-qPCR and normalized to U6 RNA level.

Supplemental Figure S3

(A) PMA-treated THP-1 cells were stimulated with 500μM of ODN2216, 1 μg/ml of CL097, and polyI:C (addition [2 μg/ml] to culture medium [100 μg/ml]) for 24 hr. The expression of mRNA was determined by RT-qPCR and normalized to GAPDH (n = 3).

(B) PMA-treated THP-1 cells were pre-treated with IFN-γ and then stimulated with 1 μg/ml of CL097 and polyI:C (addition [2 μg/ml] to culture medium [100 μg/ml]). 24 hr after stimulation, the expression was determined by RT-qPCR and normalized to GAPDH.

(C) Total RNA was extracted from EVs released from HuH-7 with or without HBV, and the expression of miR was determined by RT-qPCR and normalized to U6 RNA level. Fold increase of miR expression was calculated by dividing miR level of HBV sample by that of mock.

(D) PMA-treated THP-1 cells were transfected with miR-29a mimic for 48 hr, and then stimulated with IFN-γ, 1 μg/ml of CL097, and polyI:C (addition [2 μg/ml] to culture

medium [100 μg/ml]) for 24 hr. IL-12p40 mRNA levels were determined by RT-qPCR and normalized to GAPDH. The data is a representative of two independent experiments.

Supplemental Figure S4

(A) HBV RNA levels in HepG2-NTCP cells infected with infectious HBV particles for 14 days and then treated with or without 10 ng/ml of IFN-γ every two days.

(B) 10 μg of HBV plasmid were hydrodynamically injected into mice via tail vain. At one and three day post infection, 10 μg of mouse IFN-γ and/or 400 μg of erlotinib were intraperitoneally injected into these mice. Five days after the hydrodynamic injection, serum HBsAg levels were determined using ELISA.

Data are presented as mean ± SD (n = 3).

Supplemental Tables S1 RNA-Seq analysis of tree shrew liver after HBV infection

Supplemental Figure S1

A

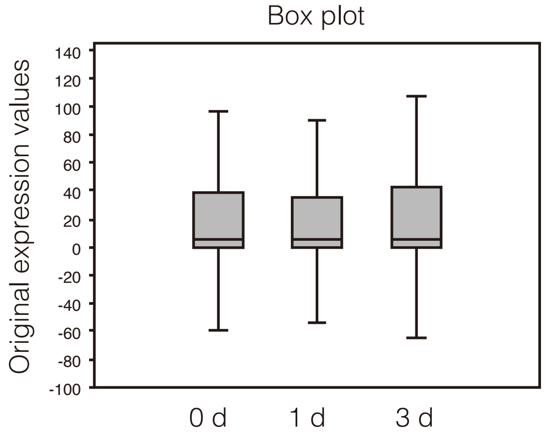

B

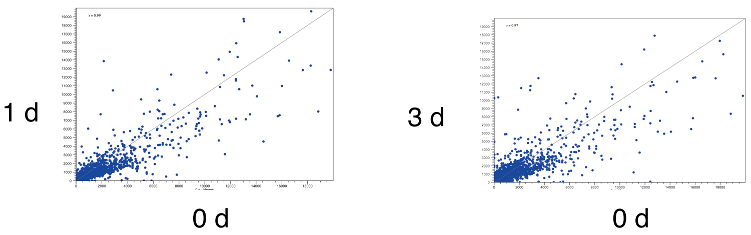

C

	0 d	1 d	3 d	
XM_006141583.1	0	1	2	CD69
XM_006168336.1	1	0	0	cGAS
XM_006168028.1	2	301	154	HLA class II histocompatibility antigen, DP alpha 1 chain-like
XM_006164174.1	0	0	0	IFN-a1
XM_006164175.1	0	0	0	IFN-a2
XM_006140329.1	0	0	0	IFN-b
XM_006147283.1	0	0	0	IFN-g
XM_006140391.1	0	0	0	IFN-l1
XM_006140390.1	0	0	0	IFN-l2
XM_006170444.1	0	0	0	IL-17A
XM_006156658.1	0	0	0	IL-1a
XM_006156657.1	0	0	0	IL-1B
XM_006153123.1	0	0	0	IL-2
XM_006156683.1	0	0	0	IL-36a
XM_006169841.1	0	0	0	IL-6
XM_006160134.1	149	101	107	IRF-3
XM_006158804.1	44	31	29	IRF-5
XM_006164434.1	676	107	194	IRF-7
XM_006163972.1	83	52	61	MAVS
XM_006160265.1	119	195	126	MDA5
XM_006156190.1	8	8	5	STING
XM_006163379.1	134	133	110	TRAF2
XM_006160032.1	0	0	0	TRAF3
XM_006165358.1	45	62	54	TRAF6
XM_006142331.1	5	15	3	Rnase L
XM_006166634.1	19	14	16	ZAP
XM_006151515.1	856	308	388	OASL
XM_006151699.1	1,524	1,252	915	OAS2
XM_006143614.1	3,593	2,448	1,973	beta-actin
XM_006170849.1	21,668	1,1503	1,0287	GAPDH

Normalized expression value

E

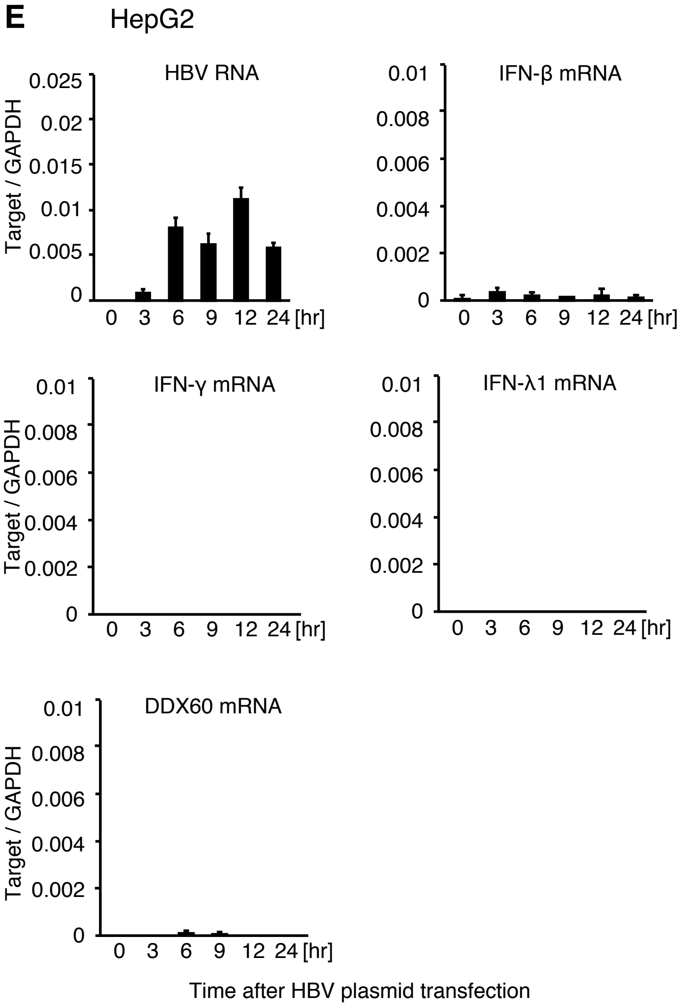

D

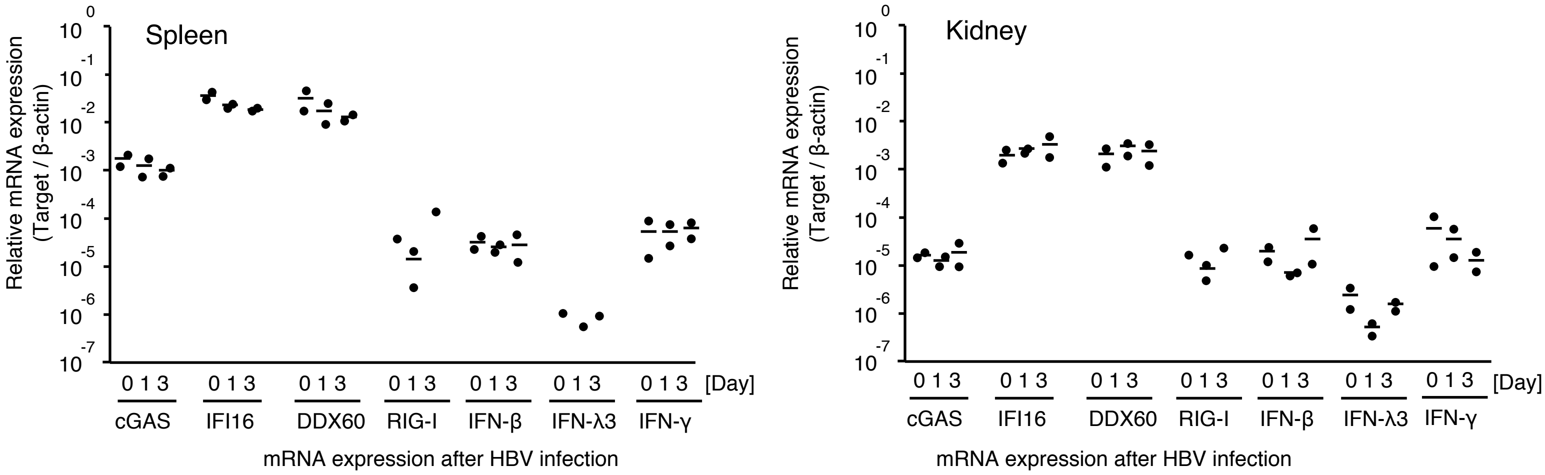

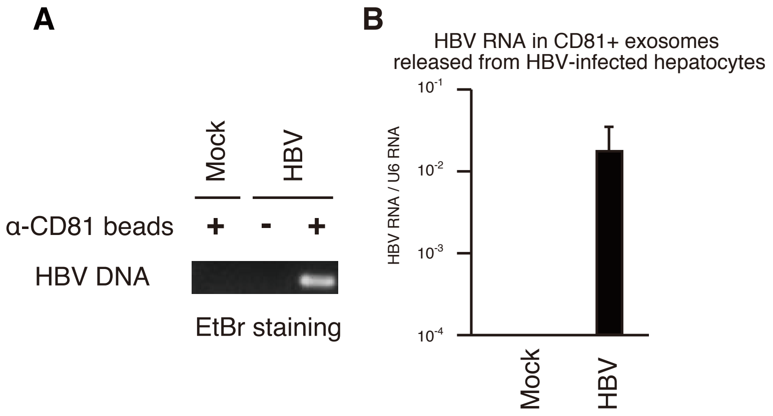
A
Mock
HBV
α-CD81 beads
+
-
+
HBV DNA
EtBr staining
B
HBV RNA in CD81+ exosomes
released from HBV-infected hepatocytes
HBV RNA / U6 RNA
10-1
10-2
10-3
10-4
Mock
HBV

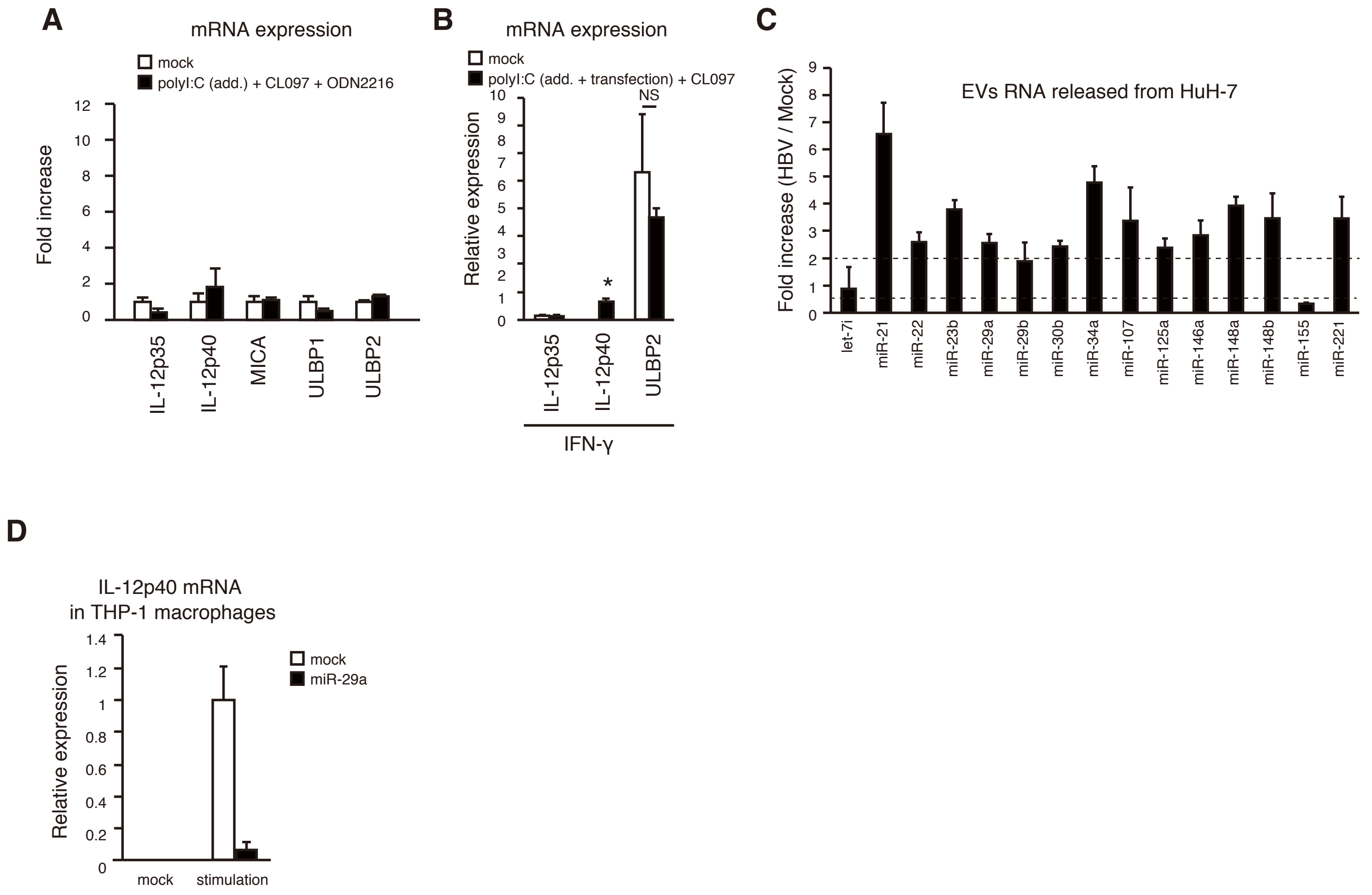
A
mRNA expression
mock
polyI:C (add.) + CL097 + ODN2216
Fold increase
IL-12p35
IL-12p40
MICA
ULBP1
ULBP2
B
mRNA expression
mock
polyI:C (add. + transfection) + CL097
Relative expression
NS
*
IL-12p35
IL-12p40
ULBP2
IFN-γ
C
EVs RNA released from HuH-7
Fold increase (HBV / Mock)
let-7i
miR-21
miR-22
miR-23b
miR-29a
miR-29b
miR-30b
miR-34a
miR-107
miR-125a
miR-146a
miR-148a
miR-148b
miR-155
miR-221
D
IL-12p40 mRNA
in THP-1 macrophages
Relative expression
mock
miR-29a
mock
stimulation

Supplemental Figure S4

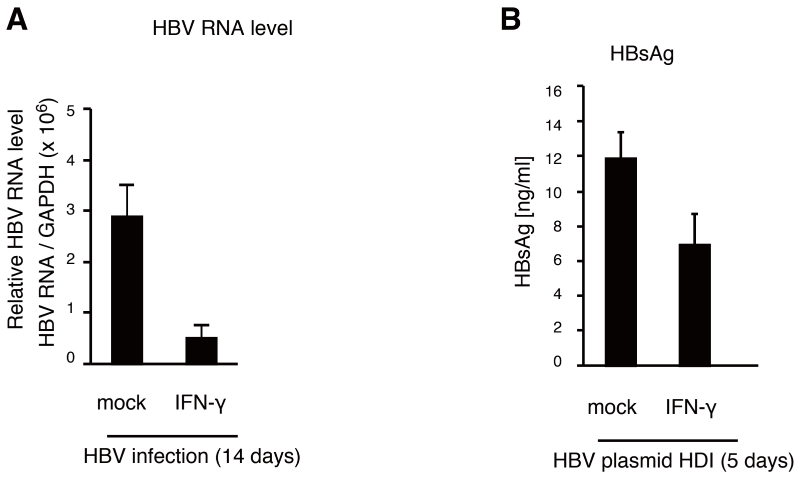

Supplement: Supplementary file 2 [file Presentation_1.PDF]
